# Preparation of Ganglioside GM1 by Supercritical CO_2_ Extraction and Immobilized Sialidase

**DOI:** 10.3390/molecules24203732

**Published:** 2019-10-16

**Authors:** Li Ji, Zhonghui Qiao, Xin Zhang, Xiaolei Cheng, Weiyang Wang, Fan Zhang, Yifa Zhou, Ye Yuan

**Affiliations:** 1 Jilin Province Key Laboratory on Chemistry and Biology of Changbai Mountain Natural Drugs, School of Life Sciences, Northeast Normal University, Changchun 130024, China; jil132@nenu.edu.cn (L.J.); qiaozh655@nenu.edu.cn (Z.Q.); chengxl811@nenu.edu.cn (X.C.); wangwy576@nenu.edu.cn (W.W.); zhangf508@nenu.edu.cn (F.Z.); zhouyf383@nenu.edu.cn (Y.Z.); 2 College of Biology and Agricultural Engineering, Jilin University, Changchun 130022, China; zhangx@jlu.edu.cn

**Keywords:** ganglioside, GM1, supercritical CO_2_ extraction, immobilization, sialidase, purification

## Abstract

Monosialotetrahexosylganglioside (GM1) has good activity on brain diseases and was developed to be a drug applied in clinics for neurological disorders and nerve injury. It is difficult to isolate GM1 in industry scale from the brains directly. In this work, a simple and highly efficient method with high yield was developed for the isolation, conversion, and purification of GM1 from a pig brain. Gangliosides (GLS) were first extracted by supercritical CO_2_ (SCE). The optimum extraction time of GLS by SCE was 4 h, and the ratio of entrainer to acetone powder from the pig brain was 3:1 (*v/w*). GM1 was then prepared from GLS by immobilized sialidase and purified by reverse-phase silica gel. Sodium alginate embedding was used for the immobilization of sialidase. Under the optimized method, the yield of high-purity GM1 was around 0.056%. This method has the potential to be applied in the production of GM1 in the industry.

## 1. Introduction

GLS is a kind of acid glycosphingolipid containing sialic acid, which is composed of sphingol, fatty acid, and oligosaccharide chain [1,2]. Gangliosides play important roles in a variety of biological processes [3,4]. Among them, GM1 can penetrate the blood–brain barrier and has very good activity on brain diseases. It is used to clinically treat neurological disorders, such as Alzheimer′s disease, Parkinson′s disease, and stroke [5,6,7,8].

GLS is widely distributed in the tissues of mammals, especially in cerebral and nervous tissue. In 1957, Folch et al. extracted total lipid extract from various tissues with the method of solvent extraction [9]. As a powerful tool, the chromatographic method is widely used to isolate and purify GLS from tissues [10], such as single-step silicic acid column [11], porous silica gel column [12], reversed-phase Lichrosorb RP8 or Bondapak RP18 column [13], DEAE–Sephadex A-25 column and latrobeads silica adsorption liquid chromatography [14], LIPSEP gel chromatography [15], and sephadex LH-20 and silica gel centrifugal liquid chromatography [16]. Although the above methods greatly promote the separation and purification of GLS from tissues, there are still several issues, including complicated operating steps, high costs, environmental pollution, and low yield. Supercritical CO_2_ extraction (SCE) has attracted much attention due to its environmental friendliness [17,18,19,20]. However, there is no report about the application of SCE in GLS extraction from the brain.

It was difficult to produce GM1 by only extraction from the brain, since the content of GM1 in the brain is very low (usually 10–20% in the total GLS) [21]. According to the structural similarities, terminal sialic acids can be removed from polysialoganglioside GD1a, GD1b, and GT1b to prepare GM1. Enzymes are catalysts bearing some excellent properties (high activity, selectivity, and specificity) that may permit to perform the most complex chemical processes. Sialidases (neuraminidases, EC 3.2.1.18) are widely distributed in living organisms and participate in the metabolism of sialoglycoconjugates. Scientists have made more efforts to convert GLS to GM1 with sialidase-producing bacteria [22,23,24,25] or recombine sialidases [26,27]. The engineering of enzymes from biological entities to industrial reactors is a very exciting goal. Fortunately, immobilization was revealed as a very powerful tool to improve almost all enzyme properties; it can also allow an easy recovery and reutilization of the enzyme. The basic methods of enzyme immobilization can be divided into five kinds: adsorption, covalent binding, embedding, microencapsulation, and cross-linking [28,29,30]. The reusable immobilized enzyme can reduce the cost of industrial production because of its effective reuse and reactor process control. Therefore, the main task of enzyme immobilization is to select an appropriate immobilization method (support, immobilization conditions, and enzyme), so as to design a method that can not only meet the catalytic needs of a certain application (productivity, stability, and selectivity), but also meet the non-catalytic needs (separation, regulation, and downstream process).

We got sialidase from *Cellulosimicrobium cellulans* sp. 21, which catalyzes the hydrolysis of α-2,3-, α-2,6-, and α-2,8-glycosidic linkages [25,27]. In this study, we established a method for extracting GLS from a fresh pig brain by SCE and chose an appropriate immobilization method to transform GLS into GM1. The purification method of the transformed product GM1 was further determined to realize the preparation of GM1 by sialidase in the industry.

## 2. Results and Discussion

### 2.1. Precipitation by Acetone and Extraction by Supercritical CO_2_

Acetone was used to remove the large amount of water (the content was >70%) and the water-soluble impurities in the fresh pig brain before the extraction. Three batches of dry acetone powder were extracted from the pig brain; the average extraction yields of the acetone powder was 22.6%. The classic method of GLS extraction is to directly extract from fresh tissue with chloroform–methanol (4:1, *v/v*). This method requires a large amount of chloroform, which is not environmentally friendly. Therefore, we used supercritical CO_2_ extraction instead of chloroform–methanol to extract GLS from acetone powder. Bao et al. extracted ganglioside from the lipid-soluble fraction of sika deer antler via supercritical CO_2_ extraction technology as follows: co-solvent 75% ethanol, extraction temperature 70 °C, and extraction pressure 30 MPa [31]. We verified that this condition was suitable for the extraction of gangliosides from the pig brain (Figure S1). In order to adapt to industrial production, we further optimized the extraction time and the volume of entrainer.

**Figure 1:**
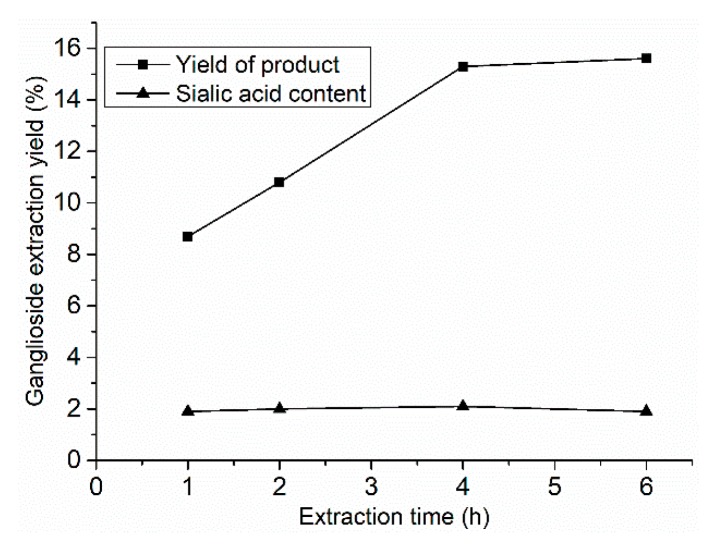
Ganglioside extraction kinetics of SCE.

The ganglioside extraction kinetic curves of SCE are shown in Figure 1. The extraction times were 1, 2, 4, and 6 h, with 120 mL entrainer (75% ethanol), at 70 °C and 30 MPa extraction pressure. The ganglioside extraction yield increased with extraction time until it peaked at 4 h, while, the content of sialic acid in the extracted product did not change with the extraction time, and was about 2%.

Thirty grams of acetone powder was extracted for 4 h with different volumes of entrainer (60, 90, and 120 mL), and the other experimental conditions were the same as the extraction kinetics of SCE. As seen in Table 1, the yields of ganglioside with 90 and 120 mL entrainer were similar, but GLS were not fully extracted with 60 mL of entrainer. Similarly, the volume of the entrainer did not affect the content of sialic acid in product.

In summary, the optimized extraction conditions were as follows: the suitable extraction time of GLS from acetone powder by SCE was 4 h, the ratio of entrainer (75% ethanol) to acetone powder was 3:1 (*v/w*), the extraction temperature was 70 °C, and the pressure was 30 MPa. Under the suitable conditions, the yield of the extracted product was 15%, and the sialic acid content was 2%.

### 2.2. Separation of Ganglioside

When ethyl acetate–methanol was used as the eluent for silica gel column chromatography, dry-sample (the sample was dissolved with a small amount of solvent, a small amount of silica gel was added, mixed well, and then spun away the solvent) loading method was used to increase the sample quantity, and 128 mg ganglioside was obtained in the eluting phase of ethyl acetate–methanol (1:1, *v/v*) from a 2 g crude sample. The ganglioside yield was 6.4%, and the sialic acid content was 20.1%, in which the content of GM1 was 11.1%, GD1a was 20.8%, and GT1b was 9.7%. Compared with the classic method, the yield and composition of gangliosides were almost the same. But, by optimizing the eluting system, ion-exchange column chromatography can be eliminated, and the preparation process can be accelerated. In the selection of eluting system, the same purification effect as chloroform–methanol can be obtained by using ethyl acetate–methanol system, as ethyl acetate is a low toxic reagent, which can reduce pollution in the process of large-scale purification and is more suitable for the preparation of gangliosides.

### 2.3. Immobilization of Sialidase

**Figure 2:**
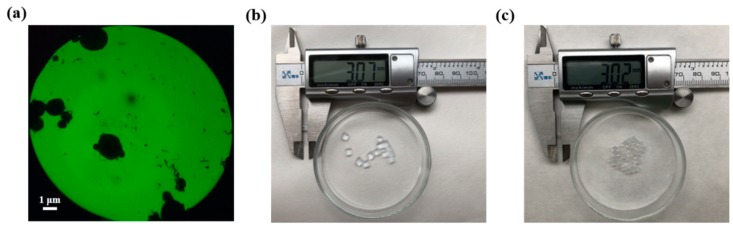
The form of immobilized sialidase with different methods: (**a**) the TEM image of magnetic nanoparticles Fe_3_O_4_–CS; (**b**) acrylamide embedding; and (**c**) sodium alginate embedding.

As sialidase CcSia was efficient in terms of reaction rate and GM1 production [27], CcSia was selected for immobilization studies. In this study, four kinds of particles were prepared, and sialidase was immobilized onto these supports with different methods. (1) Immobilization with glutaraldehyde to magnetic Fe_3_O_4_–CS at pH 5.0: The preliminary experiments showed that the maximum immobilized amount of sialidase was achieved with sialidase/Fe_3_O_4_–CS (3:10, *w/w*), and, below this weight ratio, sialidase could be 50% immobilized on Fe_3_O_4_ nanoparticles. Based on the analyses of TEM, the magnetic nanoparticles with a mean diameter of 2 μm and the particle size and structure were not affected by the immobilization process (Figure 2). (2 and 3) Immobilized sialidase was prepared using the embedding method with acrylamide–bisacrylamide and sodium alginate, respectively. (4) Sodium alginate embedding–cross-linking method was used to immobilize sialidase with CS. As shown in Table 2, the immobilized amount of sialidase reached a maximum of 95%, using the acrylamide–bisacrylamide embedding method.

### 2.4. Conversion of the Ganglioside Mixture to GM1 by Using Immobilized Sialidase

The immobilized enzyme can react continuously and simplifies the purification process, but the enzyme activity may be lost during immobilization. The desialylation activity of immobilized sialidase with four different methods was determined by suspending the beads with 30 mg sialidase in a 20 mL reaction mixture containing 10 g/L GLS in acetate buffer (pH 5.0). Reactions were performed in duplicates followed by the filtration method for biocatalyst recycling and detected by TLC.

**Figure 3:**
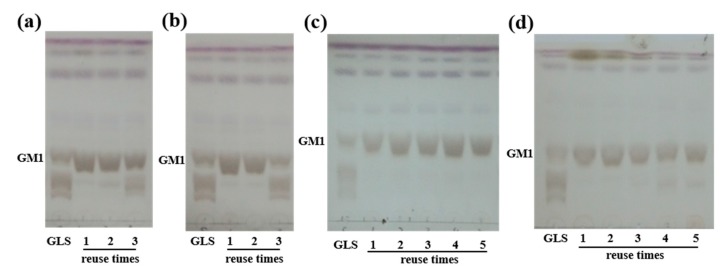
HPLC analysis of the product from GLS by immobilized sialidase with four different methods: (**a**) Fe_3_O_4_–CS; (**b**) acrylamide embedding; (**c**) sodium alginate embedding; and (**d**) sodium alginate embedding and cross-linking.

Magnetic nanoparticles have large specific surface areas and can be uniformly dispersed in the reaction system. There is almost no mass transfer resistance in the reaction system, and it can be easily separated and recycled under the action of applied magnetic field, among which the covalent cross-linked enzyme of magnetic nanoparticles has the best stability. Although we have successfully prepared magnetic nanoparticles and immobilized the sialidase by glutaraldehyde, GLS cannot be completely converted to GM1 when reused for the third time (Figure 3a), possibly because of the protein shed or decreased activity.

The embedding method is simple and can be used in industrial production. Sodium alginate, acrylamide, gelatin, etc. are commonly used as embedding agents. It could be seen in Figure 3b that the immobilization rate of acrylamide entrapped protein is very high, but it could only be reused twice, which could be caused by the leakage of sialidase. When the embedding agent was replaced with sodium alginate, it could be reused 5 times (Figure 3c), but only 44% sialidase could be fixed. The fixed amount of sialidase increased after glutaraldehyde was added, but according to TLC result (Figure 3d), the conversion was not complete after the immobilized sialidase was reused 4 times. Glutaraldehyde increased the embedding efficiency, but it may affect the activity of sialidase.

Therefore, the most suitable method for immobilization of sialidase is sodium alginate embedding: the concentration of sodium alginate and carboxymethylcellulose sodium were 1.6% and 0.2%, respectively; the concentration of sialidase was 10 mg/mL.

Previously, we used the response surface methodology to optimize GM1 production conditions. The optimum conditions for a 2 h transformation were found to be a temperature of 32.5 °C and a pH of 5.2 [27]. Under the same reaction conditions, GM1 was prepared by transforming the extracted GLS with immobilized enzyme in this work. Following biotransformation, the yield of product was 85.7%. In order to further reduce the cost, the catalytic conditions of immobilized enzyme can be optimized in the future work.

### 2.5. Purification of GM1

It is difficult to isolate and purify GM1. In order to obtain high-purity GM1, multistep chromatography was used and the detection was done using TLC. A large amount of chloroform was used in the purification process, which caused environmental pollution. The tedious purification steps and detection methods increased the production cost of GM1 and reduced the production efficiency. In this study, ion exchange, silica gel, and reverse-phase silica gel were used to purify GM1 (Table 3).

**Figure 4:**
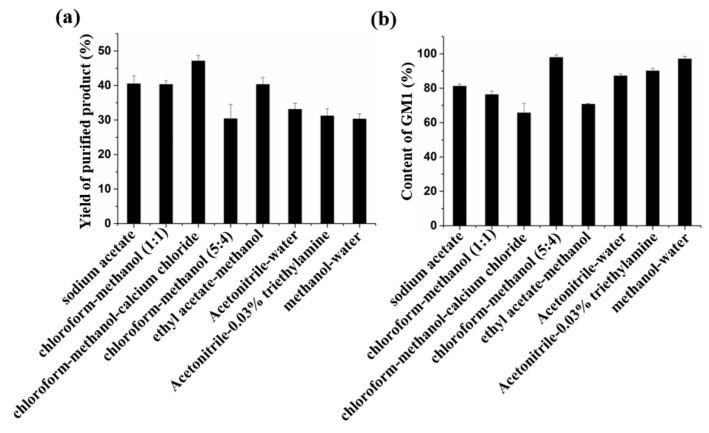
HPLC analysis of the yield and purification of GM1 by different methods. (**a**) The yield of GM1. (**b**) The purification of GM1.

In the study of ion exchange, the sample could not be combined with the DEAE-Cellulose chromatography column. TLC analysis showed that GM1 was obtained by eluting fraction in distilled water, so purification was not possible. In DEAE-Sepharose Fast Flow method, GM1 could not be eluted with 1 M NaCl. According to the properties of gangliosides, GM1 forms molecular groups in aqueous solution, and a large number of anions are exposed to the outside of the molecular cluster. Because of its strong electronegativity, it cannot be separated from ion exchange column in 1 M NaCl solution. Moreover, we found that NaCl has obvious interference in TLC detection, so GM1 is not suitable for purification by DEAE-Sepharose Fast Flow. For DEAE-Sephadex A-25, the results of HPLC analysis showed that the recovery of GM1 eluted by the chloroform–methanol–CH_3_COONa system was about 40%, and the content of GM1 was about 67%. Meanwhile, the GM1 recovery with methanol–CH_3_COONa elution system was about 37%, and the content of GM1 was 81% (Figure 4). Although DEAE-Sephadex A-25 can be used for the purification of GM1, the purity of the product is lower than that of GM1 in drugs.

For the chloroform–methanol eluting system, we used four eluents. Among them, chloroform–methanol (5:4, *v/v*) has the best separation effect, the purity of GM1 in the product was more than 97%, which meets the purity requirements for drugs, but a large amount of chloroform was needed in the preparation process. In addition, the TLC method must be used, so there are some problems in the process of large-scale preparation, such as serious pollution and difficult monitoring. In the Ethyl acetate–methanol system, although ethyl acetate–methanol (5:4, *v/v*) could elute GM1 and avoid the use of methanol, the purity is only 70.8% (Figure 4), and the loading quantity of the sample was low, so it was not suitable for the preparation of GM1.

GM1 has a UV absorption at 205–230 nm wavelength, but sodium acetate and chloroform also have absorption; chloroform has the largest interference. The interference of acetonitrile and methanol in UV detection experiment is low, so these two eluting systems can be detected by UV. In this work, acetonitrile–water (8:2, *v/v*), acetonitrile–0.03% triethylamine solution (8:2, *v/v*), and different proportions of methanol–water were used as eluents. We found GM1 was eluted in methanol–water (9:1, *v/v*) and could be detected online by UV (Figure S2). As shown in Figure 4, the yield of GM1 purified by reverse-phase silica gel column with three eluents was 30%; among them, the purity of methanol–water eluting product was 97% (Figure S3). At the same time, the use of acetonitrile was avoided.

Using the optimized method (Table S1), 0.56 g high purity GM1 (more than 97%) could be obtained from 1 kg of the fresh pig brain, which is 2.5 times the quality of GM1 obtained by direct extraction [21], and the production efficiency of GM1 is greatly improved.

## 3. Materials and Methods

### 3.1. Materials

A pig brain was provided by Jilin Qijian Bio-pharmaceutical CO., Ltd., Jilin, China. CO_2_ gas (>99.5 vol%) was obtained from Changchun Juyang Gas Co., Ltd., Jilin, China. Standards of GM1, GD1a, and GT1b were purchased from Sigma-Aldrich (St. Louis, MO, USA). Iron oxide (nanopowder), chitosan, acrylamide, *N,N′*-methylene diacrylamide, carboxymethylcellulose sodium, and sodium alginate were purchased from Aladdin (Shanghai, China). All the chemicals and reagents used were analytical grade.

### 3.2. Apparatu

Supercritical CO_2_ extraction was experimentally performed with a supercritical fluid extraction system (Newport Scientific, Inc., Jessup, MD, USA). The extraction column of this equipment has a capacity of 845 mL.

Quantitative determination was performed on a high-performance liquid chromatograph (HPLC) LC 20 AT (Shimadzu, Tokyo, Japan) equipped with an autosampler (Model SIL-20A, Shimadzu, Tokyo, Japan). The outlet was connected to a UV–VIS detector SPD 20A (Shimadzu, Tokyo, Japan) controlled by an alite system controller model CBM-20A.

### 3.3. Analytical Methods

Resorcinol–hydrochloric acid was used to identify the cerebroside. In this work, the *N*-acetylneuraminate standard sample was prepared by distilled water at 0.1 g/L. The color reagent solution was prepared as follows: 10 mL 20 g/L of resorcinol, 80 mL of HCl, and 0.25 mL 0.1 mol/L of CuSO_4_, and then the mixed solution was diluted to 100 mL with distilled water and stored at 4 °C in a brown bottle. The standard solution or sample solution was first mixed with 2 mL of the color reagent solution and heated in boiling water for 15 min. Then, the mixed solution was cooled down, and 4 mL isoamyl alcohol was added. After centrifugation, the upper organic phase was taken to measure at 620 nm [32].

The hydrolytic products were determined by thin layer chromatography (TLC). A chloroform–methanol–0.02% calcium chloride aqueous solution (60:36:8, *v/v/v*) was used as the developing agent. The gangliosides developed on the plate were stained by sulphuric acid–ethanol (5:95, *v/v*) and then incubated at 115 °C for 15 min [32].

Gangliosides were analyzed by HPLC using a Shimadzu HPLC system with an analytic Inertsil NH_2_ column (DIKMA 4.6 × 250 mm, 5 μm, Dikma Technologies, Inc., Lake Forest, CA, USA). The eluent was as follows: (A) acetonitrile: 5 mM phosphate buffer (pH 5.6) = 83:17 (*v/v*); and (B) acetonitrile: 20 mM phosphate buffer (pH 5.6) = 50:50 (*v/v*). It was eluted at the flow rate of 1 mL/min with the following gradient program: 0–15 min, 15% A; 15–30 min, 15–20% A; 30–34 min, 20% A; 34–50 min, 20–30% A; and 50–70 min, 15% A, monitored by the absorbance at 215 nm [33]. Injection volume and concentration of the sample solution were 10 μL and 1 mg/mL, respectively.

### 3.4. Precipitation of Fresh Pig Brain by Acetone

Cerebral tissue (1 kg) was homogenized with 3 L ice acetone (−20 °C) for 15 min. The homogenate was centrifuged at 3500 rpm for 10 min, and the precipitate was collected by repeated centrifugation after the addition of twice the volume of ice acetone. The precipitation was placed in a vacuum dryer at room temperature for 48 h. The powder was collected and stored for further use.

### 3.5. Supercritical CO_2_ Extraction

Supercritical CO_2_ extraction was experimentally performed with an SFE instrument (Newport Scientific, Inc., Jessup, MD, USA). The Acetone powder (30 g) was placed in the extractor, followed by delivering the entrainer by a HPLC pump (Scientific Systems, Inc., mode LS Class, Woburn, MA, USA). CO_2_ was pressurized in the compressor (Newport Scientific, Inc., Model superpressure, Jessup, MD, USA) and pumped into the extractor.

The method of Bao et al. was first verified. The effects of ethanol-modified SCE of ganglioside were performed at fixed operating conditions of 30 MPa, 70 °C, and 120 mL different concentrations of ethanol (55, 65, 75, 85, and 95 vol%). The effects of temperature-modified SCE of ganglioside were performed at fixed operating conditions of 30 MPa, 120 mL entrainer of 75% ethanol, and different temperatures (30, 40, 50, 60, 70, and 80 °C).

In order to adapt to industrial production, we further optimized the extraction time and the volume of entrainer. The effects of ethanol volume-modified SCE of ganglioside were performed at fixed operating conditions of 30 MPa, 70 °C, and different volumes of 75% ethanol (60, 90, and 120 mL) and statically extracted for 1, 2, 4, and 6 h. The SFE extracts with co-solvent were concentrated in vacuo and tested by resorcinol–hydrochloric acid method.

### 3.6. Isolation and Purification of Ganglioside

The purification of ganglioside has changed greatly from the classic chloroform–methanol method. Fifteen grams of silica gel was loaded into a chromatographic column (20 × 1.5 cm), and ethyl acetate–methanol solution (2:1, *v/v*) was used to equilibrate the column with double column volume. After loading, the sample was eluted with 2 times the volume of ethyl acetate–methanol (2:1, v/v) and 4 times the volume of ethyl acetate-methanol (1:1, *v/v*). The fraction was collected and freeze-dried.

### 3.7. High Cell Density Fermentation for Recombinant Sialidase Production

The high cell density fermentation for the production of sialidase in *E. coli* was done using a 5 L bioreactor, as described previously [27]. Protein concentration was quantified by the Bradford method, with bovine serum albumin as the external standard.

### 3.8. Different Preparation Methods of Immobilized Particles

Preparation of Magnetic chitosan (CS) particles: CS solution was prepared by dissolving 0.2 g of CS powder in 10 mL of 2% *v/v* acetic acid, after which 0.4% *w/v* iron oxide (nanopowder) was added to the CS, and then dispersed sufficiently by a sonicator. Forty milliliters of liquid paraffin and 4 mL of span-80 were mixed in a flask, the Fe_3_O_4_–CS mixture was extruded through a hypodermic needle, and then it was stirred for 30 min. Then Fe_3_O_4_–CS microspheres were activated under 400 μL 50% glutaraldehyde at 40 °C for 2 h, followed by washing with petroleum ether, methanol, and distilled water. Finally, the prepared particles were vacuum-freeze-dried.

Magnetic chitosan cross-linking: 14.6 mL acetate buffer (pH 5.0) solution, 2.4 mL 50% glutaraldehyde, and 0.1 g of magnetic Fe_3_O_4_–CS particles were mixed in a conical flask. The mixture was stirred at room temperature for 3 h; after that, the particles were isolated and washed three times with distilled water. Then, 3 mL sialidase was added to the mixture and was stirred at room temperature for 3 h. After that, the particles were isolated and washed three times with distilled water.

Acrylamide embedding: 12 mL of 30% acrylamide/0.8% bisacrylamide solution, 15 mL pH 5.0 of acetate buffer, and 3 mL of sialidase were mixed in a flask. After that, 100 μL of 10% ammonium persulfate and 40 μL of TEMED were added, swirled gently to mix, and then used immediately. After 40 min, the gel was cut into small pieces and rinsed with distilled water three times.

Sodium alginate embedding: 3 mL of sialidase, 12 mL of 4% sodium alginate, 3 mL of 2% carboxymethylcellulose sodium, and 12 mL of acetate buffer (pH 5.0) were mixed in a flask. The mixture was extruded through a hypodermic needle into a 2% CaCl_2_ solution, forming beads, and then it was immobilized for 0.5 h, and the beads were filtered out and rinsed with distilled water three times.

Sodium alginate embedding and cross-linking: 6 mL of Chitosan, 3 mL of sialidase, 2.4 mL of 50% glutaraldehyde stock solution, 12 mL of 4% sodium alginate, 3 mL of 2% carboxymethylcellulose sodium, and 3.6 mL of acetate buffer (pH 5.0) were mixed in a beaker.

### 3.9. Preparation of GM1 by Immobilized Enzyme

The beads and 200 mg GLS were added into 20 mL of acetate buffer (pH 5.0), in a flask, and shaken for 2 h at 34 °C. After that, the beads were separated and then washed with acetate buffer for reuse.

### 3.10. Separation and Purification of GM1

Ion exchange chromatography: DEAE-Cellulose, DEAE-Sepharose Fast Flow and DEAE-Sephadex A-25 were used for the separation and purification of GM1. Ten milligrams of conversion product was dissolved in 1 mL of distilled water and loaded on a balanced DEAE-cellulose (14 × 1.5 cm, CH_3_COO-type), DEAE-Sepharose Fast Flow (14 × 1.5 cm, Cl^−^ type), and DEAE-Sephadex A-25 (14 × 1.5 cm, CH_3_COO- type) ion-exchange chromatography column. The flow rate was 1 mL/min, and 4 mL of the eluent was collected in each tube and detected by TLC. Then, 0–1.0 M CH_3_COONa and 0–1.0 M NaCl were used for linear gradient elution in DEAE-cellulose and DEAE-Sepharose Fast Flow, respectively. Meanwhile, in DEAE-Sephadex A-25, chloroform–methanol–water (30:60:8, *v/v/v*) and 0.8 M of sodium acetate/methanol solution were used successfully as an eluent.

Silica gel chromatography: Chloroform/acetic ether–methanol (4:1, *v/v*) was used to equilibrate and elute the silica gel column. Chloroform–methanol–calcium chloride solution (60:36:8, *v/v/v*), chloroform–methanol (1:1, *v/v*), chloroform–methanol (5:4, *v/v*), and acetic ether–methanol (5:4, *v/v*) were used as an eluent, respectively.

Reverse-phase silica gel chromatography: The reverse-phase silica gel powder was immersed in 100% acetonitrile for 24 h and then loaded onto the columns. Acetonitrile–water (8:2, *v/v*) and acetonitrile–0.03% triethylamine solution (8:2, *v/v*) were selected as an eluent. At the same time, in order to avoid using toxic acetonitrile, a different proportion of methanol–water was used as an eluent, and UV was used for real-time detection.

## 4. Conclusions

A method for the extraction of gangliosides by supercritical CO_2_ was established; the suitable extraction time was 4 h at 70 °C and 30 MPa, and the ratio of 75% ethanol to acetone powder was 3:1 (*v/w*). Sodium alginate embedding was proved to be the most suitable method for the preparation of GM1 by immobilized sialidase. After conversion, the yield of high-purity GM1 around 0.056% of the fresh pig-brain tissue was obtained by reverse-phase silica gel chromatography, using methanol–water (9:1, *v/v*) as an eluent. The complete method may provide an alternative for the production of GM1 in the industry.

## Figures and Tables

**Table 1:** Effect of the volume of entrainer on ganglioside extraction by SCE.

No.	Volume (mL)	Time (h)	Weight(g)	Yield (%)	Sialic Acid Content (%)
1	60	4	2.2	7.3	1.9
2	90	4	4.5	14.8	2.1
3	120	4	4.6	15.3	2.1

**Table 2:** Parameters for immobilization.

Types of Carriers	Rate of Fixed Protein	Particle Size	Number of Reuse
Fe_3_O_4_–CS	49.7%	2 μm	2
Acrylamide embedding	95.1%	3 mm	2
Sodium alginate embedding	43.9%	2.4 mm	5
Sodium alginate embedding and cross-linking	89.6%	2.4 mm	4

**Table 3:** Optimization of purification method of GM1.

Purification Method	Eluent	Analytical Method
DEAE-Sephadex A25	0.8 M sodium acetate–methanol	TLC
silica gel	chloroform–methanol (1:1)	TLC
silica gel	chloroform–methanol–calcium chloride (60:36:8)	TLC
silica gel	chloroform–methanol (5:4)	TLC
silica gel	ethyl acetate–methanol (5:4)	TLC
reverse-phase silica gel	acetonitrile–water (8:2)	UV
reverse-phase silica gel	acetonitrile–0.03% triethylamine (8:2)	UV
reverse-phase silica gel	methanol–water (9:1)	UV

## Supplementary Materials

The following are available online. Figure S1: Results of the purification of GM1 by reverse-phase silica gel column. Figure S2: HPLC analysis of the product. Table S1: Extraction, conversion, and purification results of GM1 from pig brain.
