# Supplementary material for: Preparation of Ganglioside GM1 by Supercritical CO2 Extraction and Immobilized Sialidase
Source: Molecules. 2019 Oct 16;24(20):3732. doi: 10.3390/molecules24203732 (PMC6832980; doi:10.3390/molecules24203732)
Supplement: Supplementary file 1 [file molecules-24-03732-s001.pdf]

molecules

MDPI

Supplementary Materials

Preparation of Ganglioside GM1 by Supercritical CO_2 Extraction and Immobilized Sialidase

Li Ji [1], Zhonghui Qiao [1], Xin Zhang [2], Xiaolei Cheng [1], Weiyang Wang [1], Fan Zhang [1], Yifa Zhou [1], Ye Yuan [1,*]

[1] Jilin Province Key Laboratory on Chemistry and Biology of Changbai Mountain Natural Drugs, School of Life Sciences, Northeast Normal University, Changchun 130024, China; jil132@nenu.edu.cn (L.J.); qiaozh655@nenu.edu.cn (Z.Q.); chengxl811@nenu.edu.cn (X.C.); wangwy576@nenu.edu.cn (W.W.); zhangf508@nenu.edu.cn (F.Z.); zhouyf383@nenu.edu.cn (Y.Z.)

[2] College of Biology and Agricultural Engineering, Jilin University, Changchun 130022, China; zhangx@jlu.edu.cn

* Correspondence: yuany268@nenu.edu.cn; Tel.: +86-0431-85098212

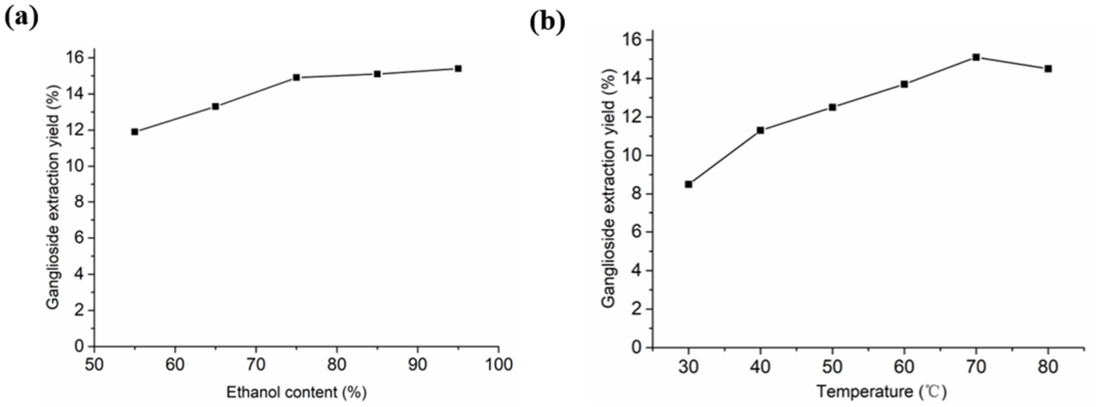

Figure S1. Effects of co-solvent concentration (**a**) and temperature (**b**) on ganglioside extraction yield.

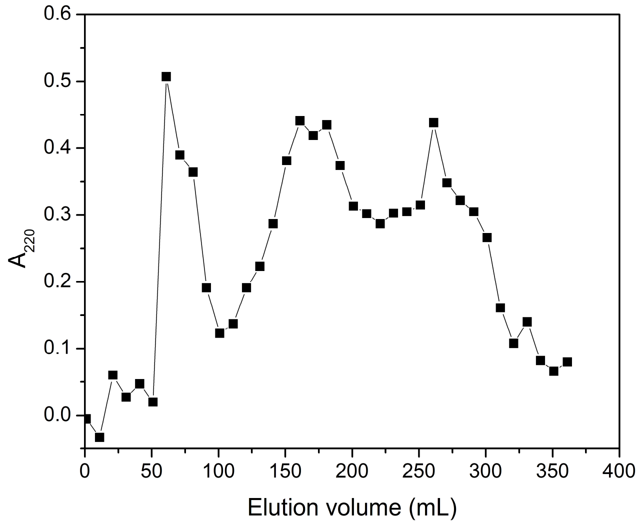

Figure S2. Results of the purification of GM1 by reverse-phase silica gel column.

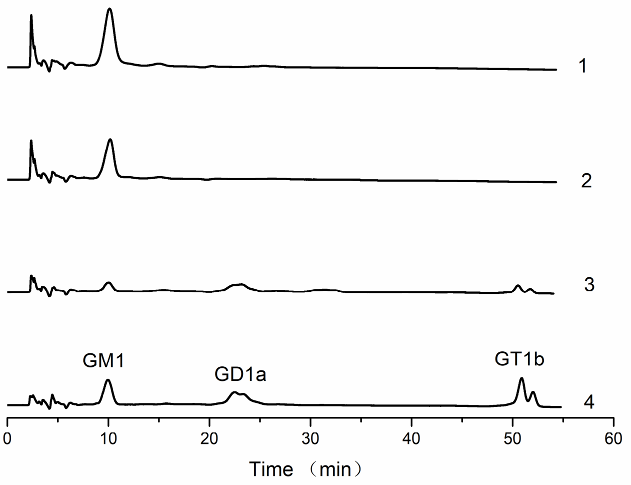

Figure S3. HPLC analysis of the product. (1) purified product by reverse-phase silica gel column; (2) Conversion product by immobilized sialidase; (3) Extraction product by SCE; (4) standards.

Table S1. Extraction, conversion and purification results of GM1 from pig brain.

Method	Weight (g)	Yield (%)
Fresh pig brain	1000	100
Dry acetone powder	226	22.6
Lyophilized powder by SCE	33.4	14.8
Lyophilized powder after silica gel column	2.1	6.4
Conversion product	1.8	85.7
Purified GM1	0.56	31.1

© 2019 by the authors. Licensee MDPI, Basel, Switzerland. This article is an open access article distributed under the terms and conditions of the Creative Commons Attribution (CC BY) license (http://creativecommons.org/licenses/by/4.0/).
